# Porous Biodegradable Metals for Hard Tissue Scaffolds: A Review

**DOI:** 10.1155/2012/641430

**Published:** 2012-07-24

**Authors:** A. H. Yusop, A. A. Bakir, N. A. Shaharom, M. R. Abdul Kadir, H. Hermawan

**Affiliations:** Medical Implant Technology Group (MediTeg), Faculty of Health Science and Biomedical Engineering, Universiti Teknologi Malaysia, 81310 Johor Bahru, Malaysia

## Abstract

Scaffolds have been utilized in tissue regeneration to facilitate the formation and maturation of new tissues or organs where a balance between temporary mechanical support and mass transport (degradation and cell growth) is ideally achieved. Polymers have been widely chosen as tissue scaffolding material having a good combination of biodegradability, biocompatibility, and porous structure. Metals that can degrade in physiological environment, namely, biodegradable metals, are proposed as potential materials for hard tissue scaffolding where biodegradable polymers are often considered as having poor mechanical properties. Biodegradable metal scaffolds have showed interesting mechanical property that was close to that of human bone with tailored degradation behaviour. The current promising fabrication technique for making scaffolds, such as computation-aided solid free-form method, can be easily applied to metals. With further optimization in topologically ordered porosity design exploiting material property and fabrication technique, porous biodegradable metals could be the potential materials for making hard tissue scaffolds.

## 1. Introduction

One of the most attractive subjects in tissue engineering is the development of a scaffold, a three-dimensional porous solid structure that plays a key role in assisting tissue regeneration [1]. Ideally, a scaffold must be porous, bioactive, and biodegradable and possess adequate mechanical properties suited to the biological site. Sufficient porosity is needed to accommodate cell proliferation and differentiation, which will eventually enhance tissue formation [2, 3]. It is also desirable for a scaffold to have high interconnectivities between pores for uniform cell seeding and distribution, and for the nutrients and metabolites exchange at the cell/scaffold construct [4–6]. A bioactive scaffold promotes cell-biomaterial interactions, cell proliferation, adhesion growth, migration, and differentiation. It also promotes extracellular matrix (ECM) deposition and permits transportation for nutrient and gases and waste removal for cell survival [2]. A biodegradable scaffold allows the replacement of biological tissues via physiological extracellular components without leaving toxic degradation products. Its degradation rate should match the rate of new tissue regeneration in order to maintain the structural integrity and to provide a smooth transition of the load transfer from the scaffold to the tissue [3]. Finally, as a mechanical support, a scaffold must possess adequate mechanical stability to withstand both the implantation procedure and the mechanical forces that are typically experienced at the scaffold-tissue interface and does not collapse during patient's normal activities [3]. Mechanically, the major challenge is to achieve adequate initial strength and stiffness and to maintain them during the stage of healing or neotissues generation throughout the scaffold degradation process [3, 7, 8].

Biodegradable polymers have been widely used and accepted as the most suitable materials for scaffolds due to their degradability, biocompatibility, and ease of processability [9–11]. Synthetic biodegradable polymers such as poly(lactic acid) (PLA), poly(glycolic acid) (PGA), and their copolymers have been used in many clinical applications [12–16]. Biodegradable polymers degrade through hydrolysis process and are gradually absorbed by the human body thus allowing the supported tissue to gradually recover its functionality [8, 17]. Biodegradability can be imparted into polymers through molecular design with a controlled rate in concert with tissue regeneration [18–21]. For instance, PLA could be combined with PGA to form poly(lactic-*co*-glycolic acid) (PLGA), which has degradation rate tailored with the tissue healing period and has been shown to support osteoblast cells attachment and growth in vitro and in vivo [22–24]. Beside copolymerization, polymer composites have been explored in order to improve mechanical property and biocompatibility. Zhang and Ma have developed [25] a highly porous biodegradable polymer/apatite composite scaffold (95% porosity) through a thermally induced phase separation technique, which resulted in significant improvement in mechanical properties compared to polymer-only scaffold. The work by Ma et al. [26] has shown that osteoblast survival and growth were significantly enhanced in the PLLA/HA composite scaffolds compared to the plain PLLA scaffolds.

**Figure 1:**
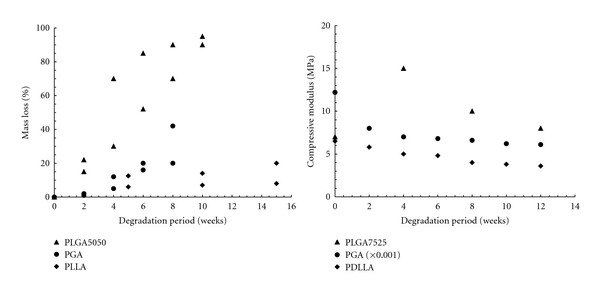
Mass loss and strength retention of some polymers used for scaffolds. Data compiled from [42–49].

One of the major concerns regarding the use of biodegradable polymers as scaffold is their poor mechanical properties [27]. For hard tissue applications such as bone, a scaffold that possesses adequate strength and Young's modulus is desirable. However, porous polymeric structures are relatively weak and may not achieve sufficient level of the required strength [8, 27]. During degradation, polymers could suddenly lose their mass and mechanical integrity.  Figure 1 illustrates mass loss and strength retention as the function of degradation period for some biodegradable polymers used for scaffold.

There is a recent and fast-growing interest in the use of biodegradable metals for biomedical applications [28]. The inherent strength and ductility owned by metals are the key features that make them appealing for hard tissue applications. Magnesium- (Mg-) based and iron- (Fe-) based metals have been used, which include Mg-RE (rare earth elements) [29–33], Mg-Ca- [34, 35], pure Fe [36, 37], Fe-Mn alloys [38, 39], and Fe foam for bone replacement scaffold [40]. Mg and its alloys have been proposed for orthopaedic implants due to their supportive physical properties to human bones. It has a density closer to that of natural bones (1.8–2 g/cm^3^) and has been reported to support the activation of bone cells [41]. Mg degrades in vivo through electrochemical process, which produces Mg hydroxide and hydrogen gas. Combining their excellent mechanical properties and degradability, Mg and its alloys are now viewed as a potential alternative for making scaffold for tissue regeneration application. Therefore, this paper aims to review the potentiality of porous biodegradable metals as material for hard tissue scaffold. Elaborations to their rationale, structure, mechanical properties, degradation, and fabrication method are presented.

## 2. Porous Mg as Scaffold Material

### 2.1. Rationale

Mg is largely found in bone tissue, it is an essential element to human body, and its presence is beneficial to bone growth and strength [52–54]. It is a cofactor for many enzymes and serves as stabilizer of DNA and RNA structures [55]. With approximately half of the total estimated 25 g content stored in bone tissue, Mg is the fourth most abundant cation in the human body [56, 57]. In the extracellular fluid, the level of Mg ranges between 0.7 and 1.05 mmol/L, and its homeostasis is maintained by the intestine and kidneys [52, 53]. The incidence of hyper-Mg is rare due to the efficient excretion of the element in the urine [52, 56].

Mg can be considered as osteoconductive and bone growth stimulator material as suggested by many studies. A significant increase of bone area has been observed in Mg-based implants compared to those based on PLA [41, 58]. The corrosion layer around Mg implants has been observed to contain calcium phosphates, which appeared to be in direct contact with the surrounding bone [41]. Xu et al. have shown [59] new bone formation around Mg-Mn-Zn implants in their in vivo degradation in rats. Witte et al. observed [60] that 3 months postoperatively, open porous Mg scaffolds implanted in rabbits were largely degraded, foreign body giant cells phagocytizing the remaining corrosion products were rarely found, and no osteolytic changes were found around the implant site. It has been shown that porous Mg has better degradation behavior in terms of lower pH change, slower hydrogen evolution, and slower decrement of compressive yield strength in simulated body fluid (SBF) immersion tests [61]. Zreiqat et al. reported [62] an increasing bone cell adhesion on Mg-enriched alumina as expressed by enhanced level of a5b1 integrin receptor and collagen extracellular matrix protein. Two studies using Mg-enriched apatites or collagen materials showed good biocompatibility on bone cell attachment and tissue growth [63, 64].

Mg and its alloys are very lightweight metals having density ranging from 1.74 to 2.0 g/cm^3^, which is less than that of Ti alloys (4.4–4.5 g/cm^3^) and is close to that of bone (1.8–2.1 g/cm^3^) [65]. They have a wide range of elongation and tensile strength from 3% to 21.8% and from 86.8 to 280 MPa, respectively. Mg possess a greater fracture toughness compared to that of ceramic biomaterials, and its elastic modulus (41–45 GPa) is closer to that of the bone compared to other metals. This property could play a vital role in avoiding the stress shielding effect. Mg also has better ductility than synthetic hydroxyapatite and higher strength than existing biodegradable polymers [66].  Table 1 shows the mechanical properties of pure Mg compared to other metals and to bones. The elastic modulus of pure Mg is closer to that of cortical and cancellous bones, which is a superior feature for bone scaffolds. Mechanical property of Mg can be further improved by alloying and thermo-mechanical processes. Addition of alloying elements such as aluminium, silver, indium, silicon, tin, zinc, and zirconium could improve both the strength and elongation of Mg alloys [67]. Moreover, some manufacturing processes such as hot rolling, hot extruding, and equal-channel angular pressing (ECAP) could also contribute to the strength of Mg alloys and in some cases also improve ductility [67–69].

### 2.2. Degradation Behavior of Mg

In physiological saline environment, Mg and its alloys degrade through the following electrochemical (corrosion) process [65, 74]:

(1) $$\begin{array}{c} \text{Mg}\left(\text{s}\right)+2{\text{H}}_{2}\text{O}\to \text{Mg}{\left(\text{OH}\right)}_{2}\left(\text{s}\right)+{\text{H}}_{2}\left(\text{g}\right) \end{array}$$

(2) $$\begin{array}{c} \text{Mg}\left(\text{s}\right)+2{\text{Cl}}^{-}\left(\text{aq}\right)\to {\text{MgCl}}_{2} \end{array}$$

(3) $$\begin{array}{c} \text{Mg}{\left(\text{OH}\right)}_{2}\left(\text{s}\right)+2{\text{Cl}}^{-}\left(\text{aq}\right)\to {\text{MgCl}}_{2}+2{\text{OH}}^{-}\left(\text{aq}\right) \end{array}$$

In the first reaction, gray Mg(OH)_2_ film is developed on the surface of Mg as it reacts with water and hydrogen bubbles are also produced. The metal can also directly react with chloride ions to form Mg chloride (2). This highly soluble MgCl_2_ is also formed through the reaction of Mg(OH)_2_ with chloride ions, as depicted in (3) [75]. Unfortunately, pure Mg corrodes very quickly in physiological solution. This may cause Mg implant to lose its mechanical integrity before the tissue is completely healed. Moreover, its corrosion reaction produces hydrogen gas at a rate that is too high to be dealt with by the host tissue [41, 76, 77]. This issue, along with the development of stainless steels in 1920s [41] led to the abandonment of Mg in spite of some early successful implantation results [76, 78, 79].

As the science and technology of Mg processing advances, many improvements have been reported to corrosion resistance as well as to mechanical properties of its alloys. As an example, Stroganov et al. reported [80] that Mg alloyed with 0.4–4 wt% REs, and other trace elements such as Cd and Al, had a slowed corrosion rate, where 3 mm diameter pins resided for 5 months, and those 8 mm in diameter resided for 11 months in vivo.  Table 2 summarizes some reports on various treatments to Mg and its alloys for biomedical applications.

### 2.3. Porous Structure of Mg

Some early studies have shown the necessity for a porous structure in bone regeneration. Kuboki et al. have shown [89] that direct osteogenesis had taken place in the porous particles of hydroxyapatite rather than the solid particles in rat ectopic model. Titanium implants recovered from sheep tibiae showed enhanced cortical shear strength from porous titanium coating, while further coating with hydroxyapatite beads did not result in significant improvement [90].

Although porosity will diminish the bulk properties of a material, porous Mg still has the strength and stiffness in close range to that of native bone. The effects of porosity and pore size on the mechanical properties of porous Mg scaffolds have been investigated [91, 92] and the results indicated that yield, compressive, and flexural strength as well as Young's modulus decreased with both the pore volume, and size. Pore morphology, volume and size can significantly affect the mechanical properties of porous Mg materials [91, 93, 94]. However, this is not critical for Mg scaffolds since their mechanical properties are still comparable to bone [95, 96]. The lower limit of bone strength is about 3 MPa [97], whilst the compressive strength and Young's modulus of cancellous bone are 0.2–80 MPa and 0.01–2 GPa, respectively [98]. Hence, the range of bone stiffness and strength may be achieved by Mg scaffolds by modulating their porosity and pore sizes. Porosity also eventually decreases corrosion resistance of porous Mg. Zhuang et al. had evaluated [92] in vitro degradation behavior of two different Mg porosities in physiological saline solution (0.9% NaCl). The specimens with 55% porosity degraded faster than those with 36% porosity due to more connecting areas and transport channels for the solution to perform faster chemical reactions.

Porous architecture of Mg scaffold has been proven to play a significant role in cell growth and proliferation. Tan et al. reported [50] their work on three-dimensional open-cellular Mg structure fabricated by mechanical perforation method. By using the Taguchi method, they concluded an optimum pore configuration at 70% porosity, 300 *μ*m pore size, and 90° pore arrangement angle whereas these three parameters had different effects on the compressive properties. Pore distribution also influences rabbit cranial bone ingrowth behavior as proved by Simon et al. [99]. They observed a continuous ingrowth in the random pore size scaffolds from the outer periphery; meanwhile, for the same sized pores and solid walls scaffolds, discontinuous ingrowth with bone islands throughout the whole scaffold was observed.

### 2.4. Fabrication of Porous Mg

In the recent years, synthesis of cellular metals having open or closed pores of either periodic or random pore topology has been extensively studied. Periodic structure offers advantages over random architectures in designing better mechanical efficiency and function [100]. There have been various routes to modulate the periodic structure topology to satisfy a range of applications including biomedical implants [101].

**Figure 2:**
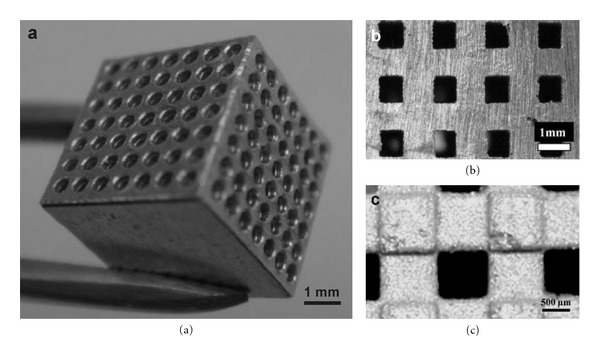
Example of porous Mg scaffolds: (a) made by laser-assisted mechanical perforation technique, adapted with permission from IOP [50]; (b, c) made by solid free-form fabrication method, adapted with permission from John Wiley and Sons [51].

Random cellular Mg can be fabricated via powder or chip sintering (conventional, laser assisted, or spark plasma), low pressure casting, or removable spacer methods. These fabrication routes generate a random cell structure, wide distributions of cell size, and morphology leading to unpredictable material properties over the range of hundred microns [102, 103]. Processes that can be used to fabricate Mg with topologically ordered open cell structure include solid free-form process, space holder method, leaching method, replication, electrodeposition, and vapor deposition.  Figure 2 shows example of porous Mg scaffolds made by two different processes.

#### 2.4.1. Solid Free-Form Process

This technique encompasses rapid prototyping and casting processes, where a step-by-step fabrication process from computer-aided design (CAD) models is adopted. It is currently an ideal solution for manufacturing complex 3D porous structures by accurate controlling of the structure topology [104]. The six basic steps in the synthesis of a topologically ordered porous Mg (TOPM) are (1) creating a 3D model with the desired architecture using CAD; (2) printing a positive polymeric template of the model by rapid prototyping (RP) process; (3) infiltrating the polymeric template with a NaCl paste; (4) removing the template by heating followed by sintering of NaCl; (5) casting liquid Mg into NaCl template, that is, with pressure assistance; (6) removing NaCl template by dissolution [51, 105].

This technique gives several advantages including the ease of providing considerable high surface area of scaffold, which is essential for cell growth and cell proliferation: also no toxic solvent is involved in the process. Staiger et al., one of the pioneers in this technique, achieved [105] high replication accuracy with a resolution of 0.8 mm and errors of 5~12% for their porous Mg. Kirkland et al. reported [106] their capability to create square pores with a size of at least 0.3 mm × 0.3 mm, and Witte et al. produced [107] open porous Mg scaffolds with 72 to 76% porosity and pore sizes ranging between 10 and 1000 *μ*m, and tested into the patellar cartilage of rabbits where new bone formation was observed.

Precautions should be taken during the removal process of NaCl from the cast Mg structure. The remaining salt will dissolve into aqueous solution and aggressive chloride ions will increase Mg corrosion [108–110]. Hence, only fresh NaOH solution should be used to dissolve NaCl to reduce the buildup of chloride ions. Overinfiltrating, partially infiltrating, and underinfiltrating should be avoided so as to obtain a perfect replicate casting of 3D CAD model [51]. It should be noted also that molten Mg at high pressure will infiltrate not only pores of the NaCl mould, but also the micropores between sintered NaCl particles, resulting in the impossibility of the removal of all residual NaCl from the structure.

Challenge in this technique includes dimensional change from polymer model to NaCl template to Mg structure. Nguyen et al. reported [51] dimensional change between 0.3 and 0.4 mm for strut sizes converted from RP model to NaCl template as a result of low filling efficiency during NaCl infiltration process. The difficulty to infiltrate occurred at pore sizes smaller than 0.8 mm. It was also identified that the use of NaCl is limited by the strength or fragility of the sintered NaCl template, which then limits casting capability. The use of larger NaCl particle distribution size (i.e., from 20 to 65 mm) has been suggested to improve the flow of NaCl paste and the strength of sintered template. Resolution of the RP printer is also another limitation to produce finer structures at a size similar to the hierarchical structure of human bone.

#### 2.4.2. Space Holder, Leaching, and Other Potential Methods

The limitation in obtaining homogenous pore size by powder metallurgical method can be solved by the use of space holder materials [91–93]. As an example, carbamide (CO(NH_2_)_2_) has been used as a spacer material to fabricate porous structures of pure Mg [92]. The process produced open-cellular Mg (porosities: 36–55%; pores: 200–400 *μ*m) having mechanical properties close to that of natural bone. The leaching method has been applied to produce porous Mg-calcium-phosphate (MCP), where macropores and micropores were created by NaCl particles and saturated NaCl solution, respectively [111]. Other potential methods are electrodeposition and vapor deposition. Electrodeposition involves the use of metallic ions solution to deposit metallic elements on electrically conductive polymeric foam [112]; meanwhile vapor deposition uses chemical reactants in gaseous phase, which were heated by radiation prior to their deposition on polymeric precursor substrate [113].

## 3. Porous Fe as Scaffold Material

Fe is an essential element that plays significant roles in human body metabolism including transport, activation, and storage of molecular oxygen, reduction of ribonucleotides and dinitrogen [114], and decomposition of lipid, protein, and DNA damages [115]. Fe has a higher elastic modulus (211 GPa) compared to that of Mg (41 GPa) and its alloys (44 Gpa) and 316L stainless steel (190 Gpa) [116, 117]. Peuster et al. are among the first who proposed [36] Fe as a biodegradable metal, where they reported an in vivo implantation test of Fe stents in the descending aorta of rabbits. They showed evidence that pronounced inflammatory response and systemic toxicity were not observed up to 18 months of the study.

Currently, there is limited literature on Fe as a scaffold material. Very recently, Farack et al. have studied [40] Fe foam coated with calcium-phosphate for bone replacement scaffold where human mesenchymal stem cells proliferated and differentiated more on coated Fe foams than on uncoated ones. The coating gave enhanced bioactivity and inhibited degradation of Fe foams; however, the latest is actually questionable since Fe was generally viewed as having too slow degradation for implant applications [118]. The open porous Fe and Fe-phosphorous alloys have been investigated as biodegradable bone replacement [119], and the results showed that addition of phosphorus increased compressive yield up to 11 MPa, higher than that of pure Fe of 2.4 MPa, and resulted in a Young's modulus of 2.3 GPa which is comparable to that of typical bone. The alloys showed also faster in vitro degradation than pure Fe but still considered slow as large fraction of material was observed during 12 months in vivo study [119]. Nevertheless, alloying Fe with phosphorous seems to be a promising way to optimize both mechanical and degradation properties of Fe especially for bone scaffold.

Porous Fe has been fabricated via several methods including solid-gas eutectic solidification process [120, 121], CO-CO_2_ gas foaming powder metallurgy process [122], or powder metallurgy with the use of polymer foaming agent [119, 123], or even using wood as template [124]. However, those techniques hardly provide the topologically ordered porous as desired for bone scaffold. Moreover, owing to its very high melting temperature, the solid free-form method as applied to Mg seems to be nonapplicable for Fe where the excessive heat might destruct the NaCl template.

## 4. Perspective

Biodegradable metals as tissue scaffolding materials have been viewed as alternative to polymers for hard tissue regeneration exploiting mostly their superior mechanical properties over biodegradable polymers. Biodegradable metals such as Mg and its alloys possess mechanical properties in close range to those of native human bone and have shown encouraging results when used as tissue scaffolds. Porous Fe could also be viewed as a potential scaffold material but available data is scarce especially in its relation to bone tissue. Among many promising techniques to fabricate metal scaffolds, solid free-form is currently viewed as the most potential method to fabricate biodegradable metal scaffolds having optimized pore morphology for cell growth and cell proliferation. This technique permitted the design and realization of topologically ordered porous Mg with periodic structure for enhanced mechanical efficiency and function of a porous scaffold. Further investigations are needed in the solid free-form fabrication method to develop a scaffold with properties specifically tailored for cell regeneration and tissue growth.

Overall, application of biodegradable metals for tissue engineering scaffold is just in the beginning. Limited work has been done and much has still is to be done. The directions could be in finding suitable process for making porous structure from all prospective biodegradable metals, understanding the influence of porous structure to mechanical and degradation properties, and understanding the cell regeneration and degradation product transport in the porous structure. Integrating biodegradable polymers or ceramics and drugs could be another interesting direction to explore.

## Figures and Tables

**Table 1:** Mechanical properties of Mg compared to bone and metals.

Tissue/material	Density (g/cm^3^)	Ultimate tensile strength (MPa)	Yield strength (MPa)	Elastic modulus (GPa)
Cortical bone [70]	1.8–2.0	35–283	104.9–114.3	5–23
Cancellous bone [70]	1.0–1.4	1.5–38	—	10–1570 (MPa)
Ti6Al4V [70]	4.43	830–1025	760–880	114
316L stainless steel [71]	8.0	450–650	200–300	190
Pure Mg, annealed [72]	1.74	160	90	45
WE43 Mg alloy, T6 [73]	1.84	220	170	44

**Table 2:** Reports on corrosion resistance of Mg and its alloys.

Material and method	Findings
Calcium addition to AZ91Ca (1 wt%) and AZ61 (0.4 wt%) alloys [81]	The high amount of Ca in the alloy enhanced the formation of calcium phosphate on the surface and improved corrosion resistance; there was only a slight decrease in mechanical property of the alloy in SBF as compared to that of in air
Mg-Mn and Mg-Mn-Zn alloys [82]	Addition of Mn and Zn elements accelerated the formation of Mg-containing phosphate and provided better protection for matrix alloy; Zn-containing phosphate layer provided an effective protection to the alloy
Alkaline heat treatment on Mg-Ca alloy [83]	Corrosion rates of treated alloy in SBF were decreased; the treated alloy samples did not induce toxicity to L-929 cells during 7 days of culture
MgF_2_ coating on extruded LAE442 alloy [84]	Extruded LAE442 alloy provided low corrosion rates and reacts in vivo with an acceptable host response; localized corrosion attack was observed in both coated and uncoated LAE442 implants
Hydroxyapatite coating on AZ91 alloy [85]	The coated alloy showed 20% improvement in the mechanical strength as compared to that of the uncoated one; 40% loss in the mechanical strength after 5 days of exposure to SBF was measured for the uncoated alloy
Hydroxyapatite coating with MgF_2_ interlayer on pure Mg [86]	Coated Mg corroded less than bare Mg and showed an effective protection from in vivo corrosion; coated Mg had a higher bone-to-implant contact ratio in the cortical bone area of the rabbit femora 4 weeks after implantation
Phosphating treatment to form brushite layer on extruded Mg-Mn-Zn alloy [81, 87]	Electrochemical and immersion tests showed that the brushite (CaHPO_4_·2H_2_O) coating provided a good protection against corrosion in SBF; corrosion resistance increased with the increase of the phosphating time within 50 min
Chitosan coating on Mg-1Ca alloy [88]	Corrosion resistance of the coated alloy in SBF was improved
